# Live Imaging of a Hyperthermophilic Archaeon Reveals Distinct Roles for Two ESCRT-III Homologs in Ensuring a Robust and Symmetric Division

**DOI:** 10.1016/j.cub.2020.05.021

**Published:** 2020-07-20

**Authors:** Andre Arashiro Pulschen, Delyan R. Mutavchiev, Siân Culley, Kim Nadine Sebastian, Jacques Roubinet, Marc Roubinet, Gabriel Tarrason Risa, Marleen van Wolferen, Chantal Roubinet, Uwe Schmidt, Gautam Dey, Sonja-Verena Albers, Ricardo Henriques, Buzz Baum

**Affiliations:** 1 MRC-Laboratory for Molecular Cell Biology, UCL, Gower Street, London WC1E 6BT, UK; 2 Molecular Biology of Archaea, Institute of Biology II - Microbiology, University of Freiburg, 79104 Freiburg, Germany; 3 Center for System Biology Dresden (CSBD), 01307 Dresden, Germany; 4 Max Planck Institute of Molecular Cell Biology and Genetics (MPI-CBG), 01307 Dresden, Germany; 5 Institute for the Physics of Living Systems, UCL, London WC1E 6BT, UK; 6 Gratentour, France; 7 Figeac, France

**Keywords:** ESCRT-III, live-cell imaging, cell division, DNA segregation, archaea, *Sulfolobus acidocaldarius*, thermophiles, hyperthermophiles

## Abstract

Live-cell imaging has revolutionized our understanding of dynamic cellular processes in bacteria and eukaryotes. Although similar techniques have been applied to the study of halophilic archaea [1, 2, 3, 4, 5], our ability to explore the cell biology of thermophilic archaea has been limited by the technical challenges of imaging at high temperatures. *Sulfolobus* are the most intensively studied members of TACK archaea and have well-established molecular genetics [6, 7, 8, 9]. Additionally, studies using *Sulfolobus* were among the first to reveal striking similarities between the cell biology of eukaryotes and archaea [10, 11, 12, 13, 14, 15]. However, to date, it has not been possible to image *Sulfolobus* cells as they grow and divide. Here, we report the construction of the *Sulfoscope*, a heated chamber on an inverted fluorescent microscope that enables live-cell imaging of thermophiles. By using thermostable fluorescent probes together with this system, we were able to image *Sulfolobus acidocaldarius* cells live to reveal tight coupling between changes in DNA condensation, segregation, and cell division. Furthermore, by imaging deletion mutants, we observed functional differences between the two ESCRT-III proteins implicated in cytokinesis, CdvB1 and CdvB2. The deletion of *cdvB1* compromised cell division, causing occasional division failures, whereas the *ΔcdvB2* exhibited a profound loss of division symmetry, generating daughter cells that vary widely in size and eventually generating ghost cells. These data indicate that DNA separation and cytokinesis are coordinated in *Sulfolobus*, as is the case in eukaryotes, and that two contractile ESCRT-III polymers perform distinct roles to ensure that *Sulfolobus* cells undergo a robust and symmetrical division.

## Highlights

• Development of a heating chamber for live imaging of hyperthermophiles
• Live imaging of cell division in the archaeon *Sulfolobus acidocaldarius*
• DNA reorganization is tightly coupled to cell division in *Sulfolobus acidocaldarius*
• ESCRT-III homologs play distinct roles in ensuring robust and symmetric division

## Graphical Abstract

**Figure:**
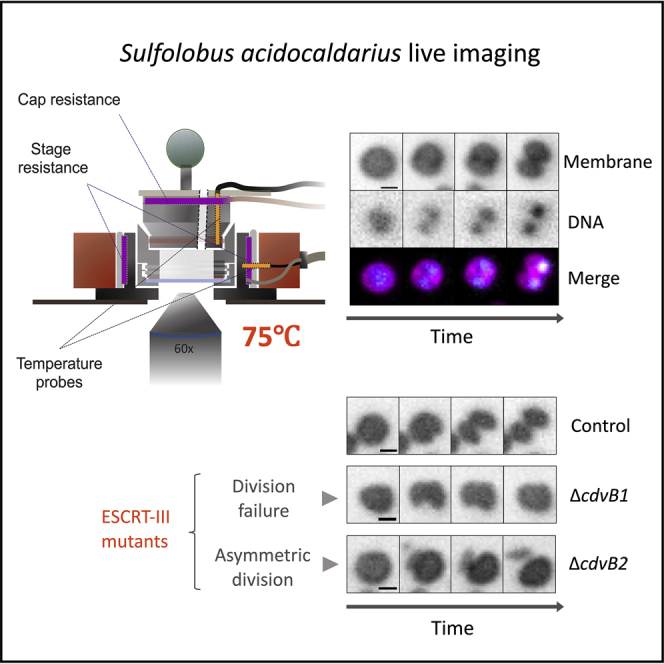

## Teaser

Pulschen et al. describe the “Sulfoscope,” a microscopy platform that allows live imaging of hyperthermophiles. By imaging cell division in the archaeon Sulfolobus, they observe tight coupling between DNA reorganization and cytokinesis and complementary roles for two ESCRT-III homologs in ensuring robust and symmetric cytokinesis.

## Results

### High-Temperature Live Imaging of Fluorescently Labeled *Sulfolobus* Cells

**Figure 1:**
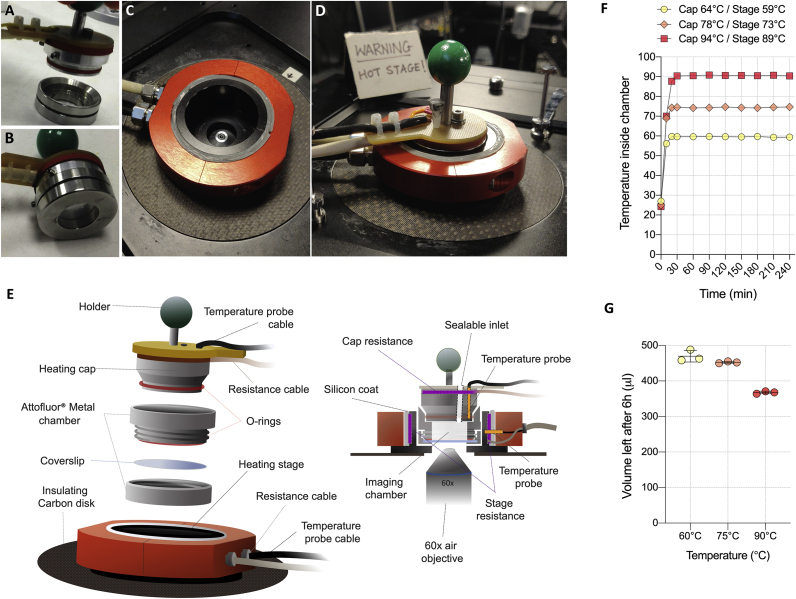
Heating Elements and Characterization of the *Sulfoscope* (A) Heating cap and Attofluor chamber, disconnected. (B) Assembled cap with Attofluor chamber. (C) Heating stage view from the top. (D) Fully assembled system. (E) Schematic of the heating system. (F) Temperature measurements performed inside the system. Temperature was recorded using a probe inserted into the imaging chamber through the sealable inlet. (G) Evaporation measurements after 6 h at the desired temperature. Error bars show mean and SD.

In order to achieve the stable high temperatures (70°C–80°C) required for live imaging of thermophilic archaea, like *Sulfolobus acidocaldarius*, we designed a chamber consisting of two individual heating elements: a cap and stage (Figures 1A–1E). Our first-generation design employed a heated stage and a metal chamber (Figure 1A, bottom part). However, this led to large (>10°C) temperature gradients across the chamber because of the poor conductivity of the glass coverslip and thermal losses to the surrounding room-temperature air. Furthermore, the open top led to the rapid loss of water from the chamber through evaporation. This could not be resolved using a simple seal, because this resulted in evaporated water condensing onto the underside of the lid. To overcome both problems, inspired by PCR machines, we designed and built a heated cap for the chamber (Figures 1A and 1E), which could be sealed using an O-ring (Figure 1B). To further improve the temperature control, we custom-built a heated stage (Figures 1C and 1E) that perfectly fit the chamber and cap (Figure 1D). By applying a temperature gradient, so that the cap was raised to a temperature 5°C higher than the base, the medium quickly reached a stable equilibrium temperature that could be maintained for long periods of time with minimal loss of water (Figures 1F and 1G).

**Figure 2:**
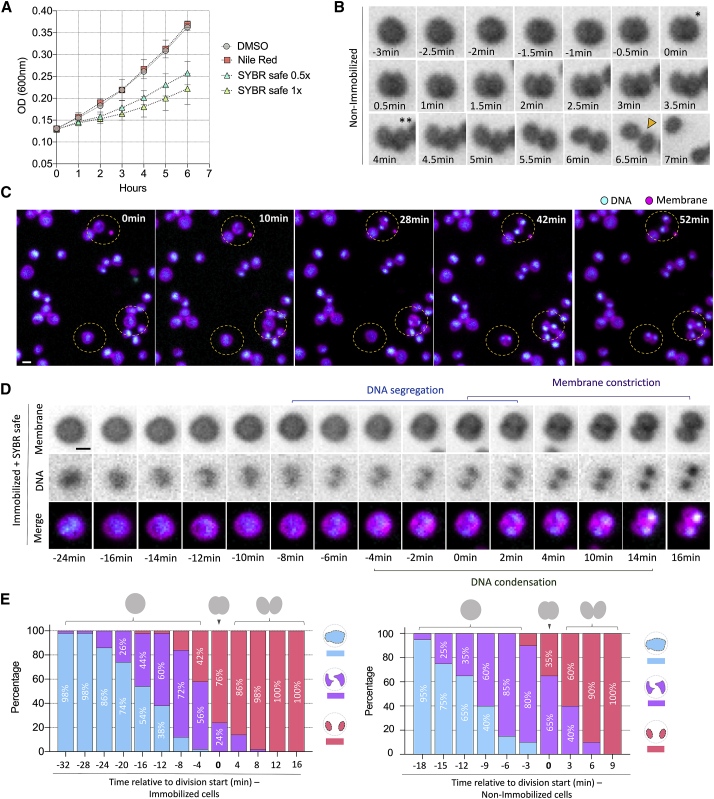
Live-Cell Division of *Sulfolobus acidocaldarius* DSM 639 (A) Growth curve of *S. acidocaldarius* treated with Nile Red, SYBR Safe, and control. Error bars show mean and SD. (B) Time-lapse of a non-immobilized *Sulfolobus* cell stained with Nile Red alone. ^∗^ = start of cytokinesis. ^∗∗^ = end of cytokinesis, orange arrowhead = cell separation. (C) Time-lapse microscopy showing immobilized cells segregating their DNA and dividing. (D) Time-lapse imaging of an immobilized cell as it divides, showing changes in the membrane and DNA organization. (E) Changes in DNA organization that accompany division in immobilized cells (n = 50) and non-immobilized cells (n = 20). Cells were separated into three different classes based upon their DNA organization: Cells with a single diffuse structure (blue), two diffuse structures (purple), or compact and well-defined structures (pink). Scale bars: 1 μm. Error bars show mean and SD. See also Figures S1 and S2 and Videos S1 and S2.

In order to image *Sulfolobus acidocaldarius* cells live using this setup, cells were pre-labeled using dyes (Nile Red for membrane and SYBR Safe for DNA) that retain their optical properties at high temperature and low pH. Cell immobilization proved the greater challenge. Although *Sulfolobus* cells could be imaged without immobilization in heated chambers, only a small number of cells remained static long enough to allow for accurate quantitative measurements to be made. Additionally, to be sure that observed changes in DNA reorganization during division were not due to cell movement, cells had to be held in place. Unlike bacteria cells, however, *Sulfolobus* cells appear to be soft and sensitive to mechanical stress (Figure S1D)—in line with observations made in other archaea [1, 2]. So, to provide a soft support sufficient to prevent cells from moving, we placed cells under a semi-solid, preheated Gelrite pad (see STAR Methods for details). We identified conditions under which it was possible to combine this soft immobilization with dyes and two-color fluorescent imaging to follow *S. acidocaldarius* cells for up to 2 h, after which cell divisions under these conditions became rare. Whereas the membrane dye proved non-toxic, the DNA dye, as reported for many other cells, reduced the rate of cell growth (Figure 2A). Therefore, where possible (e.g., for the study of division symmetry and failures), measurements were performed using Nile Red alone. Comparisons of cell division rates under these different conditions can be found in Figure S1. The fastest division times were recorded for cells imaged in the absence of a DNA dye without immobilization—conditions closest to those found in liquid culture (Figure 2B; Figure S1).

### Live Imaging Reveals Coordination of DNA Segregation, Compaction, and Cytokinesis in Dividing *Sulfolobus acidocaldarius* Cells

Using the Sulfoscope, we were able to assess the dynamics of events accompanying cell division in the thermophilic archaeon *Sulfolobus acidocaldarius*. In these experiments, *S. acidocaldarius* cells were found to be near spherical and to divide to generate two oval daughter cells (Figures 2B–2D). Imaging also revealed coordinated changes in DNA organization and cell division (Videos S1 and S2). The first evidence that cells were about to divide was a change in DNA organization prior to the start of membrane furrowing (Figure 2D). During this period, the DNA underwent a transition from a diffuse state to a semi-compact state in which the nucleoids appeared partially separated (Figure 2E). The onset of furrowing was accompanied by further compaction of the DNA, leading to the formation of two distinct, spatially separated chromosomes (Figures 2C–2E). Importantly, we observed the exact same order of events and the same changes in DNA changes over time in the presence or absence of a Gelrite pad (Figure 2E; Figure S1C). Reassuringly, a similar sequence of events was also seen in movies of dividing *Saccharolobus solfataricus* cells (formerly *Sulfolobus solfataricus* [16]) (Figures S2A and S2B), imaged at 75°C using the same setup, suggesting that the process is conserved.

Video S1. Cell Division of *Sulfolobus acidocaldarius* DMS639 sta#ined with Nile Red (membr#ane) and SYBR safe (DNA), Related to Figure 2

Video S2. Field of Cell of *Sulfolobus acidocaldarius* DMS639 Stained with Nile Red (Membrane) and SYBR Safe (DNA), Showing Several Examples of Divisions, Related to Figure 2

### The Role of the Contractile ESCRT-III Proteins, CdvB1 and CdvB2, in *S. acidocaldarius* Cell Division

**Figure 3:**
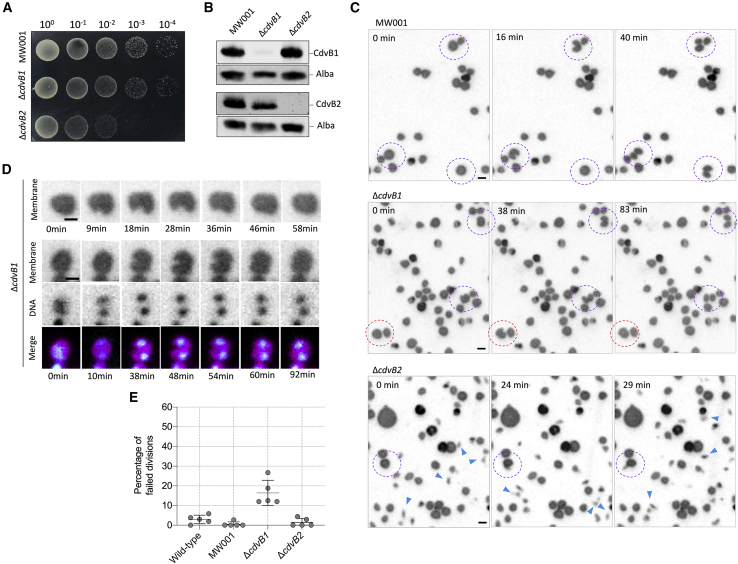
Effects of the Deletion of *cdvB1* and *cdvB2* in Cell Division and Cell Growth in *Sulfolobus acidocaldarius* (A) Growth of MW001 (background strain), Δ*cdvB1*, and Δ*cdvB2* on BNS medium plates, showing that the growth of Δ*cdvB2* is compromised. (B) Western blots of MW001, Δ*cdvB1*, and Δ*cdvB2* using CdvB1 and CdvB2 antibodies. The DNA binding protein Alba was used as a loading control. (C) Time-lapse microscopy showing MW001, Δ*cdvB1*, and Δ*cdvB2* cells. Purple dashed lines encircle cells undergoing successful divisions, whereas cells undergoing failed divisions are encircled by red dashed lines. The blue arrowheads point to small cells that drift across the imaging field. (D) Time-lapse imaging of Δ*cdvB1* cells that following membrane furrowing fail division, resulting in the fusion of daughter cells. (E) Quantification of the frequency of division failures in immobilized wild-type, MW001, Δ*cdvB1*, and Δ*cdvB2* cells. Five independent movies were used to evaluate the frequency of division failure from 130 to 180 divisions per strain, in total. Scale bars: 1 μm. Error bars show mean and SD. All live imaging shown in this figure was performed using immobilized cells. See also Figure S2 and Videos S3 and S4.

Risa and collaborators [17] recently proposed a model of ESCRT-III-mediated division in *S. acidocaldarius* based on data acquired from fixed cells. Under this model, a non-contractile CdvB division ring is formed at the equatorial plane of dividing *Sulfolobus* cells that templates the assembly of a contractile ESCRT-III heteropolymer based on two other ESCRT-III proteins, CdvB1 and CdvB2. The sudden proteasome-mediated degradation of CdvB then triggers division by allowing the constriction of the CdvB1 and CdvB2 ring [17]. An untested assumption of this model is that CdvB1 and CdvB2 are equivalent in their ability to drive scission [17]. Although this fits with their co-localization, and is in keeping with previous studies that identified CdvB1 and CdvB2 as close paralogs that share 65% amino-acid identity and 80% similarity, we wondered if we could dissect their individual function using the *Sulfoscope*. To test this, we generated in-frame deletion mutants of *cdvB1* and *cdvB2* in the *S.* background strain MW001 and imaged these cells live (Figures 3A and 3B; Video S3). Interestingly, although growth was severely impaired in the *cdvB2* deletion strain, the deletion of *cdvB1* only had a modest impact on growth at 75°C (Figure 3A). Moreover, Δ*cdvB1* cells had an average size that was similar to cells from the background strain, MW001 (Figure 3C; Video S3).

Video S3. Fields of Three Different Strains: MW001 (Control), Δ*cdvB1*, and Δ*cdvB2*, Related to Figure 3 Several small, triangular cell can be seen moving cross the field in the Δ*cdvB2* movie. Red arrows point to failed divisions.

When imaged live, a subpopulation of Δ*cdvB1* cells failed midway through the division process (Figures 3D–3E; Video S4). In such cases, a halt in cytokinesis caused the nascent daughter of Δ*cdvB1* cells to fuse back together to generate single cells that carry both copies of the spatially separated and compact chromosomes (Figure 3D). Under our imaging conditions such failures accounted for 16% of Δ*cdvB1* divisions but were very rare (1%–2%) in the other strains analyzed (Figure 3E). However, the growth of Δ*cdvB1* colonies was much more severely impaired at 65°C (Figure S2C). At this lower temperature, we observed a significant population of >2N cells and an increase in the number of cells unable to complete cytokinesis (Figures S2D–S2H). Thus, although CdvB1 plays only a minor role in *Sulfolobus acidocaldarius* division under optimum growth conditions, it appears necessary to ensure that division remains fail-safe and is robust under suboptimal conditions.

**Figure 4:**
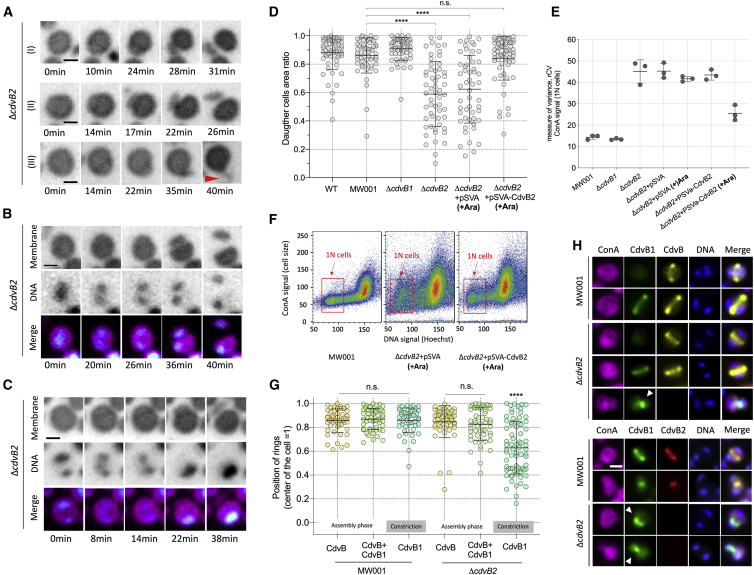
The ESCRT-III Protein CdvB2 Ensures Division Symmetry in *Sulfolobus acidocaldarius* (A) Asymmetric divisions, from moderate (I and II) to strong (III) asymmetries. Red arrowhead shows a very asymmetric cell division. (B) Asymmetric division in which both daughters inherit one copy of the genome. (C) Asymmetric division of a cell leading to the formation of a ghost cell. (D) Daughter cell area ratios for different strains. A value of 1 represents a perfect symmetric division, whereas smaller values indicate increasing asymmetry. At least three independent movies and at least 50 cells were evaluated for each strain (^∗∗∗∗^p < 0.0001, n.s., not significant. statistical test: non-parametric Mann-Whitney). (E) Variation in the size of newly divided ethanol fixed cells as estimated by flow-cytometry. The plot shows robust coefficient of variance (rCV = st.dev/median) for the ConA-alexa 647 signal, which marks the cell periphery (S-layer). (F) Plots based upon an analysis using flow cytometry indicating variation in cell size for MW001, Δ*cdvB2*+pSvA (Induced), and Δ*cdvB2+*pSvA-CdvB2 (Induced). Note that the expression of CdvB2 from a plasmid partially rescues the division phenotype. Newly divided cells have a 1N DNA content. (G) Quantification of the position of ESCRT-III division rings in fixed MW001 and Δ*cdvB2* cells that were immunolabelled for CdvB and CdvB1. A value of 1 represents the center of the cell. Ring positions (n = 50 to 60) were estimated for different stages in the division process [17] (^∗∗∗∗^p < 0.0001, n.s., not significant. statistical test: non-parametric Mann-Whitney). (H) Immunolocalization of CdvB, CdvB1, and CdvB2 in MW001 and Δ*cdvB2* cells. White arrowheads show asymmetric divisions. Scale bars: 1 μm. Error bars show mean and SD. All live imaging shown in this figure was performed using immobilized cells. See also Figure S3 and Videos S4 and S5.

Video S4. Examples of Divisions, Related to Figures 3 and 4. MW001, failed divisions - Δ*cdvB1* and asymmetric divisions - Δ*cdvB2*.

As suggested by the colony phenotype, the impact of CdvB2 loss on division was much more profound than the loss of CdvB1 at 75°C. At a first glance, this was seen by an increase in the variation in cell size in the population, as well as by the presence of small, irregularly shaped cells not properly immobilized by the pad (Figure 3C; Video S3). Upon further examination, such phenotypes were found to result from asymmetric divisions (Videos S3 and S4). Thus, the site of division in Δ*cdvB2* cells proved extremely variable (Figures 4A–4D). In most cases, each of the two daughter cells generated by an asymmetric division retained one of the two separated chromosomes (Figure 4B). However, in some cases, extreme mispositioning of the furrow led to the formation of ghost cells (Figure 4C) and a corresponding population of large cells with extra chromosomes (Figures S3A and S3B). The asymmetry was also visible as a high variance in the size of newly born G1 cells (and to a lesser extent G2 cells; Figure S3C), as indicated by the analysis of fixed cells labeled with a DNA dye by flow cytometry (Figures 4E and 4F). Importantly, both the division asymmetries and the cell size variation in Δ*cdvB2* cells could be rescued by the ectopic expression of CdvB2 from an inducible promoter (Figures 4D–4F; Figures S3D and S3E; Video S5), demonstrating that these defects are due to a lack of CdvB2.

Video S5. Field of Δ*cdvB2*+ pSVA-CdvB2 Cells (Rescue Strain) Compared to Δ*cdvB2*+ pSVA Cells (Empty Plasmid), Related to Figure 4 In the rescued strain divisions appeared symmetric and small cells were rare.

To determine the cause of the error in division symmetry in Δ*cdvB2* cells, we used immunostaining of different components of the ESCRT-III ring (CdvB, CdvB1, and CdvB2), to determine where in the previously described division sequence [17] defects in ring positioning first arise (Figure 4G). CdvB, the first component of the ESCRT-III ring to assemble during division, was found correctly positioned in the center of both MW001 and Δ*cdvB2* cells (Figure 4H). Furthermore, with few exceptions, the same was true for cells that co-stained for CdvB and CdvB1 (Figure 4G). However, following proteasome-mediated degradation of CdvB [17], while CdvB1 was correctly positioned at the cell center in the MW001 control, CdvB1 rings were found at variable positions in Δ*cdvB2* cells starting to divide (Figures 4G and 4H). These data suggested that CdvB2 is recruited (along with CdvB1) to the correct position at the cell center by the CdvB ring and functions, following the loss of CdvB, to prevent the division ring from slipping as it constricts.

## Discussion

Here, we describe the development of the “*Sulfoscope*,” an imaging platform that makes it possible to image *Sulfolobus* cell divisions live. Our first use of the *Sulfoscope* revealed a tight coupling between DNA reorganization, nucleoid separation, and membrane deformation during division (Figure 2D). These events appear to occur in a defined order in the wild-type cell (both *S. acidocaldarius* and *S. solfataricus*; Figures S2A and S2B). This begins as replicated chromosomes lose their diffuse organization as they separate and is followed by a relatively sudden DNA compaction, which coincides with the onset of furrowing. Thus, DNA compaction may help to ensure that the segregated chromosomes remain out of the way of the furrow as it closes. These findings are in line with observations in fixed cells of *Sulfolobus* [18] and are similar to DNA segregation behavior observed in fixed cells of *Halobacterium salinarium* [19].

By combining molecular genetics and live-cell imaging, we were also able to use the *Sulfoscope* to define homolog-specific roles for the two ESCRT-III proteins (*cdvB1* and *cdvB2)* that form part of the contractile division ring [17]. Unlike *cdvB*, which is an essential gene [20], *cdvB1* and *cdvB2* deletion strains could be obtained and their individual roles investigated using live-cell imaging. This analysis revealed that, although CdvB1 and CdvB2 are co-localized in the contracting division ring (Figure 4H) and are close homologs, they perform distinct functions. The loss of CdvB2 had the most striking phenotype, which was characterized by a profound failure in division symmetry. Remarkably, our analysis suggests that the wide variance in the size of daughter *ΔcdvB2* cells, which included a proportion of ghost cells and daughter cells with twice the normal DNA content (Figure 4C; Figure S3D), resulted from slippage of the CdvB1 ring as it constricted (Figures 4D and 4G). By contrast, although division failures were only occasionally observed in the corresponding *ΔcdvB1* mutant cells, these divisions remained symmetric. These data show that, though it is possible for the two proteins to form a co-polymer in the wild type, as has been suggested to be the case for many of their eukaryotic ESCRT-III counterparts [21, 22], they likely perform distinct but complementary functions in ring contraction. Our data suggest that CdvB2 fixes the contracting ring in place following the loss of the CdvB scaffold, perhaps by binding tightly to the membrane, whereas CdvB1 may be required to aid force generation to ensure that the process is fail-safe and may be particularly important under stressful conditions, e.g., following changes in temperature (which might alter the biophysics of the membrane) or mechanical resistance. Strikingly, despite these differences, our data also show that either one of the two contractile ESCRT-III rings is sufficient to drive division in many cells.

Previous work provides support for the idea that CdvB1 and CdvB2 play distinct functional roles in division. For example, an earlier study found that the deletion of *cdvB2* rendered cells more slower growing than did a *cdvB1* deletion [20]. Similarly, in other studies, *Sulfolobus islandicus* strains with reduced levels of the CdvB1 protein were found to be viable [23], as was a deletion strain [8], whereas *cdvB2* was found to be essential [8, 23]. Most notably, Liu and collaborators [23] observed distinct morphological defects when overexpressing truncated versions of CdvB1 and CdvB2 in *S. islandicus*. In this case, the truncated versions of the CdvB2 version generated cells that the authors suggested were arrested in the final stages of abscission [23], with bridges that resembled eukaryotic midbodies, whereas the overexpression of truncated CdvB1 generated populations of cells that connected cells to one another in chains. Although some of the differences in the findings in these different studies may reflect strain differences, it is clear from our analysis that live-cell imaging greatly aids the mechanistic study of dynamic processes like division.

Importantly, our findings have striking parallels in eukaryotes. In mammalian cells, for example, the two ESCRT-III proteins CHMP2A and CHMP2B, which were thought to be functional homologs, were recently found to contribute differently to membrane remodeling and to have different affinities for the membrane [24]. Moreover, in *in vitro* studies, different eukaryotic ESCRT-III proteins have been shown to work together to increase membrane binding of the heteropolymer [25]. Our data suggest that the same might apply in *Sulfolobus*, where two different ESCRT-III homologs, CdvB1 and CdvB2, act cooperatively to ensure proper membrane binding and constriction—preventing ring slippage or cytokinesis failure. We think this work also makes the case for *Sulfolobus* being a simple, well-defined system in which to study ESCRT-III-dependent division.

Finally, though we have used the *Sulfoscope* to reveal fundamental novel aspects of cell division, we anticipate that this type of high-temperature live-imaging platform can be used to shed light on other exciting areas of *Sulfolobus* cell biology, including DNA remodeling [15, 26], swimming [27], conjugation [28, 29], viral infection [23, 30], competition [31, 32], and cell-cell fusion [33]. In addition, this system can now also be applied to the study of other thermophilic microbes, from eukaryotes to bacteria [34, 35]. Although we hope that our work can open new avenues of research and lead further developments in the field of live imaging of thermophiles [36, 37], the further development of thermophile cell biology will also depend on the establishment of a thermostable fluorescent proteins [38, 39] and the use of microfluidics, to allow for the tracking of cells over long periods across multiple generations, as currently performed in Haloarchaea [40].

## STAR★Methods

### Key Resources Table

**Table:** 

REAGENT or RESOURCE	SOURCE	IDENTIFIER
**Antibodies**		

Rabbit Anti-CdvB	[17]	N/A
Chicken Anti-CdvB1	[17]	N/A
Guinea pig Anti-CdvB2	[17]	N/A
Mouse Anti-Alba	[17]	N/A
Goat anti-rabbit IRDye 680CW	LI-COR	Cat# 926-68021; RRID:AB_10706309
Donkey anti-chicken IRDye 800CW	LI-COR	Cat# 926-32218; RRID:AB_1850023
Donkey anti-guinea pig 800CW	LI-COR	Cat# 926-32411; RRID:AB_1850024
Goat anti- mouse IRDye 800CW	LI-COR	Cat# 926-32210; RRID:AB_621842
Goat anti-rabbit Alexa Fluor 546	Invitrogen	Cat# A11035; RRID:AB_143051
Goat anti-guinea pig Alexa Fluor 488	Invitrogen	Cat# A11073; RRID:AB_2534117
Goat anti-chicken Alexa Fluor 488	Invitrogen	Cat# A11039; RRID:AB_142924

**Chemicals, Peptides, and Recombinant Proteins**		

SYBR safe	Thermo Fisher Scientific	Cat# S33102
Nile red	Sigma-Aldrich	Cat #72485
Concanavalin A Alexa Fluor 647 Conjugated	Thermo Fisher Scientific	Cat# C21421
DNase I	Thermo Fisher Scientific	Cat# EN0521
EDTA-free protease inhibitor cocktail	Roche	Cat# 11836170001
Hoechst	Invitrogen	Cat# H3570

**Experimental Models: Organisms/Strains**		

*S. solfataricus* P2 (Wild type)	DSMZ	DSM1616
*S. acidocaldarius* DSM639 (Wild type)	DSMZ	DSM639
MW001 Uracil auxotrophic background strain Δ*pyrE* (Δbp 91–412), background: *S. acidocaldarius* DSM639	[6]	N/A
Δ*cdvB1 (*Saci_0451) (background MW001)	This work	N/A
Δ*cdvB2* (Saci_1416) (background MW001)	This work	N/A
Δ*cdvB2+*pSVA (Δ*cdvB2* transformed with pSVA empty plasmid)	This work	N/A
Δ*cdvB2*+pSVA-CdvB2 (Δ*cdvB2* transformed with pSVA containing the wild-type gene of CdvB2, under an inducible promoter controlled by Arabinose)	This work	N/A

**Recombinant DNA**		

Plasmid pSVA431 (Backbone of deletion plasmid containing a *pyrEF* and *LacS* cassette from *S. solfataricus)*	[6]	N/A
Plasmid pSVA1895 Used for deletion of *cdvB1* (Saci_0451)	This work	N/A
Plasmid pSVA1896 Used for deletion of *cdvB2* (Saci_1416)	This work	N/A
Plasmid pSVAaraFX-stop Backbone of expression plasmid harboring an arabinose inducible promoter containing a *pyrEF* cassette from *S. solfataricus*	[41]	N/A
Plasmid pSVAaraCdvB2 Used for expression of CdvB2 (Saci_1416)	This work	N/A

**Software and Algorithms**		

Adobe Ilustrator	Adobe Inc.	https://www.adobe.com/products/illustrator
Prism v.8	GraphPad	https://www.graphpad.com/scientific-software/prism/
ImageJ (FiJi)	NIH	https://imagej.nih.gov/ij/
ImageJ plugin StackReg	[42]	http://bigwww.epfl.ch/thevenaz/stackreg
FlowJo v10.1	FlowJo	https://www.flowjo.com/solutions/flowjo

### Resource Availability

#### Lead Contact

Further information and requests for resources, *Sulfolobus* strains and reagents should be directed to and will be fulfilled by the Lead Contact, Buzz Baum (b.baum@ucl.ac.uk).

#### Materials Availability

All unique/stable reagents generated in this study are available from the Lead Contact without restriction.

#### Data and Code Availability

This study did not generate any unique dataset or code.

### Experimental Model and Subject Details

*Sulfolobus acidocaldarius* wild-type (DSM639), *Saccharolobus solfataricus* wild-type, and mutants (*ΔcdvB2*+pSVAaraFX-stop (empty plasmid control) and *ΔcdvB2*+pSVA-CdvB2) were grown at 75°C in Brock Salts medium supplemented with 0.1% NZ-amine and 0.2% Sucrose (BNS), final pH corrected to 3.5 with H_2_SO_4_. Uracil was added to the final concentration of 20 μg/mL when working with uracil auxotrophic strains MW001, *ΔcdvB1* and *ΔcdvB2* strains. The density of liquid cell cultures was maintained at OD600nm values between 0.05 and 0.3 as measured by a spectrophotometer.

### Method Details

#### Strain constructions and growth conditions

Deletion of genes was performed using the “pSVA431 method” as previously described by Wagner and collaborators [10]. For CdvB2 overexpression, a synthetic gene containing restriction sites (NcoI and XhoI) flanking the *cdvB2* gene (Saci_1416) was used (Thermo Scientific®) and cloned into the pSVAaraFX-stop plasmid. For the *ΔcdvB2*+pSVAaraFX-stop and *ΔcdvB2*+pSVA-CdvB2 rescue experiments, expression was induced by adding 0.15% w/v (final concentration) of L-arabinose to freshly diluted cultures. Cells were grown overnight with L-arabinose and collected the next morning for live-imaging, immunofluorescence and flow cytometry, at an OD600nm of 0.1 to 0.25. The experiment was repeated three times on independent days.

#### Cap/stage construction and development

The heating system was designed to be combined with the Attofluor® chamber. The metal elements in the cap and the heating elements were built with aircraft aluminum (AL 7075 for the cap and stage and AL 2024 for the metal collars) using a lathe to obtain the final shape. Screws used are stainless steel 304. The insulating disk was built with carbon fiber. The top part of the heating cap was built using fiberglass and a silicon rubber, which separates the metal and the fiberglass and holds the electric resistance (thin film resistance). The temperature of the cap and the chamber were controlled by two independent controlling systems attached to the heating elements. The temperature used in the cap is a thermocouple type T; the one used for the chamber is a miniPT100. Detailed information about the design of the chamber, controlling system and materials is available upon request.

#### Chamber measurements

For measurements of the temperature inside the chamber, a digital thermometer with a probe (Signstek 6802 II with a 2k type probe) was used. The probe was inserted inside the chamber trough the sealable inlet (Figure 1) and positioned exactly at the middle of the chamber, touching the glass. 500 μL of Ultrapure water was added to the chamber, since in our imaging conditions, liquid media is always present. Readings were taken initially every 10 min (for the first 30 min) then followed up for 30 min. A more comprehensive follow up was also performed in which the temperature was followed continuously for half an h for every tested temperature. We never observed a temperature variation greater than 0.5°C during the duration of these measurements. For the evaporation assays, 500 μL of BNS media was added to the chamber, and the weight of the chamber was measured using an analytical scale. The pre-heated system was then closed. After six hours, the heating system was turned off. After cooling back to room temperature, the Attofluor chamber was removed and the weight was recorded again. The difference in the final weight calculated and considered to reflect the loss of liquid due to evaporation.

#### Live imaging methodology

All the manipulation of cells and materials prior to live imaging at high temperature were conducted in a way to minimize temperature loss during the transport from the incubators to the microscope. In order to achieve this, metal beads were placed inside a container that was heated overnight at 75°C. Aliquots of the cultures (5mL to 10mL) were transferred to previously clean and heated flasks placed inside the metal beads container, and then transported to the microscope. When done properly, cells suffer from minimal heat stress, and cell division can be imaged immediately after the start of the image acquisitions. The commercial Attofluor chamber and coverslips were washed thoroughly with BNS media, followed by a wash with ultrapure water before use. We noticed that dirty coverslips can damage the membrane of *Sulfolobus* cells. The assembled chamber containing the clean coverslip needs to be pre-heated before imaging starts. This can be achieved during the washing/drying steps.

For live imaging of *S. acidocaldarius* and *S. solfataricus*, Nile red (Sigma) was used at 2.5 μg/mL final concentration and SYBR safe (Thermo Fisher Scientific), 10,000x concentrated was used at a final dilution of 1x to 0.5x. Cells were imaged at an OD_600nm_ of 0.1 to 0.35. For imaging non-immobilized cells, 400ul of culture was added to the chamber. Cells were allowed to settle for 5 min before commencing image acquisition. For imaging immobilized cells, we used heated semi-solid BNS pads (0.6% Gelrite, 50% non-pH BNS, with 20mM final concentration of CaCl_2_). Pads were prepared by cutting circular (8mm) disks from a semi-solid BNS media Petri dish, prepared as described above (25mL per plate). To facilitate handling, a 13mm circular coverslip was used to support the pad after it was removed from the plate. The pads, now inside a closed Petri dish, were placed at an incubator at 75°C degrees for 20 min prior to imaging and transported inside a container full of heated metal beads (as described above) to the microscope. After loading 400 μL of the cell culture inside the chamber, the heated pads were placed on top of the cells (using forceps, holding the 13mm coverslip with the pad on top). The chamber was quickly closed and positioned for imaging. We observed that heating up the pads prior to imaging not only prevented heat shock, but also slightly dried the pads. This causes the cells to accumulate at the border of the pads once placed inside the chamber. Movies were therefore acquired within these regions where many cells can be found that are only subject to moderate levels of confinement. Imaging immobilized cells, with DNA dye, membrane dye under constant light exposure was limited to two hours, since the combined effect as found to be toxic over periods longer than this. Imaging with Nile Red only can be performed for longer periods.

#### Imaging acquisitions

Images were acquired using a Nikon Eclipse Ti-2 inverted microscope, equipped with a with a Prime 95B sCMOS camera (Photometrics). A 60x air objective (Plan Apo 60x/0.95 objective, Nikon) combined with the 1.5x additional magnification from the Ti-2 microscope body was used, resulting in an effective magnification of 90x (Pixel size = 0.1235 μm). For membrane imaging, Nile red was illuminated with 550nm LED illumination at 20% of maximum intensity, while for DNA imaging SYBR safe was illuminated with 470nm LED at 25% of the maximum intensity (pE-4000 LED illuminator, CoolLED). Exposure time was limited to about 40 ms for each channel. Whenever DNA was imaged together with Nile red, images were acquired every 2 min. For measurement of division asymmetry and failure division events, only Nile red was used, and images were acquired every 30 s to every 1 min. For correction of XY axis drifting in the images, was used ImageJ plugin StackReg [42]. Focal drift was avoided by using the inbuilt Perfect Focus System.

#### Immunofluorescence labeling

Cells were fixed sequentially in ice-cold ethanol until reaching the final concentration of 70%, starting at 30%. For the analysis, cells were centrifuged for 3min at 8,000xg and resuspended in PBSTA (PBS + 0.2% Tween20 + 1% BSA) for 5 min for rehydration. After rehydration, cells were incubated with 100 μL of primary antibodies diluted in PBSTA for 2 h, at room temperature and in a small thermomixer at 500rpm. Cells were washed twice in PBSTA and them incubated in 100μl of secondary antibodies diluted in PBSTA, with 200 μg/mL of Concavalin A conjugated with 647 Alexa Fluor for 1 h, 500rpm, at room temperature. Cells were washed twice again, resuspended in PBSTA and 1 μg/mL of Hoechst to visualize DNA.

#### Immunofluorescence microscopy

Microscopy slides were prepared by coating LabTek chambered slides with 2% polyethyleneimine (PEI) for 30 min at 30°C. Slides were then washed with distilled water and 200 μL of stained cell suspension was added and spun for 30 min at 500 - 1000 RCF in a swing-bucket rotor. Images were captured for 50ms to 100ms using a Nikon Ti2, 1.39NA 100x Objective, and Photometrics Prime 95B SCMOS camera.

#### Flow cytometry

Flow cytometry analysis was carried out on BD LSR II and BD Fortessa with immunostained cells going through the following lasers and filters. Lasers: 355 nm, 488 nm, 561 nm, 633 nm Filters: 450/50UV, 525/50 Blue, 582/15 YG, 474 710/50 Red. Side Scatter and Forward scatter was also recorded. Analysis were performed using FlowJo software v10.

#### Western blotting

*S. acidocaldarius* pellets were lysed in 100-250 μL lysis buffer (TK150 buffer, supplemented with DNase I, and EDTA-free protease inhibitor cocktail, 0.1% Triton X-100). Cells were disrupted in a sonicator (Diagenode) and subsequently centrifuged at 4°C (10,000 × g for 15 min). Supernatant was collected and protein concentration was measured using Bradford reagent (Bio-Rad). About 8-15 μg of total protein extract was resolved on NuPAGE 4%–12% Bis-Tris gels (Invitrogen) using MES SDS running buffer (Invitrogen). Proteins were transferred to a 0.45 μm nitrocellulose membrane. Membranes were blocked with PBST (PBS, 0.2% Tween20) with 5% non-fat milk. Primary and secondary antibodies were diluted in this same buffer. Membranes were incubated with primary antibodies overnight at 4°C. Primary antibodies anti-CdvB1, anti-CdvB2 and anti-Alba were used as described in Risa, 2019. Next, the membranes were washed with PBST and incubated with Li-COR IRDye 680CW or 800CW secondary antibodies diluted 1:10000 for 1 h at room temperature. Finally, membranes were washed with PBST and developed using Li-COR Odyssey Infrared Imaging System. Images were analyzed on ImageJ.

### Quantification and Statistical Analysis

All statistical tests were conducted using GraphPad Prism. Statistical test used: non-parametric Mann–Whitney. Changes in DNA organization that accompany division in immobilized cells were estimated by using 50 cells for the immobilized treatment and 20 cells for non-immobilized cells. For quantification of division failure (75°C), five independent movies were used to evaluate the frequency of division failure from 130 to 180 divisions per strain, in total. For comparison of cell division speeds and failure rates at 65°C, cells from two independent movies were used. Division time was estimated as the duration from the start of constriction until the two cells were physically separated and between 42 to 66 events were quantified for each strain, in total. For the asymmetry measurements, cells area were measured using Fiji (ImageJ). At least three independent movies and the area of at least 50 cells divisions were evaluated for each strain. For comparison of the impact of different treatments on cell division, cells were segmented using StarDist [43] and division events were detected using Trackmate [44] on these segmentation results. By fitting a sigmoidal curve to the length of the short axis of the cell over time, the period of time corresponding to division-associated morphological changes could be defined as the time between the two inflection points of the fitted sigmoid function (Figure S1E, shaded gray region on plots), using between 15 to 20 cells per treatment. Ring positions (n = 50 to 60 rings) were estimated using ImageJ, for different stages in the division process. Quantification of number of division rings per 1000 cells in MW001 and Δ*cdvB1* at 65°C was performed manually. At least 3000 cells for each strain was analyzed in total (at least 1000 from each independent triplicate) and the number of rings quantified. For correction of XY axis drifting in the images, was used ImageJ plugin StackReg [42]. Focal drift was avoided by using the inbuilt Perfect Focus System.

## Supplemental Information

Document S1. Figures S1–S3

Document S2. Article plus Supplemental Information
