# Supplementary material for: Live Imaging of a Hyperthermophilic Archaeon Reveals Distinct Roles for Two ESCRT-III Homologs in Ensuring a Robust and Symmetric Division
Source: Curr Biol. 2020 Jul 20;30(14):2852–2859.e4. doi: 10.1016/j.cub.2020.05.021 (PMC7372223; doi:10.1016/j.cub.2020.05.021)
Supplement: Document S1. Figures S1–S3 [file mmc1.pdf]

Current Biology, Volume *30*

Supplemental Information

Live Imaging of a Hyperthermophilic Archaeon Reveals Distinct Roles for Two ESCRT-III Homologs in Ensuring a Robust and Symmetric Division

Andre Arashiro Pulschen, Delyan R. Mutavchiev, Siân Culley, Kim Nadine Sebastian, Jacques Roubinet, Marc Roubinet, Gabriel Tarrason Risa, Marleen van Wolferen, Chantal Roubinet, Uwe Schmidt, Gautam Dey, Sonja-Verena Albers, Ricardo Henriques, and Buzz Baum

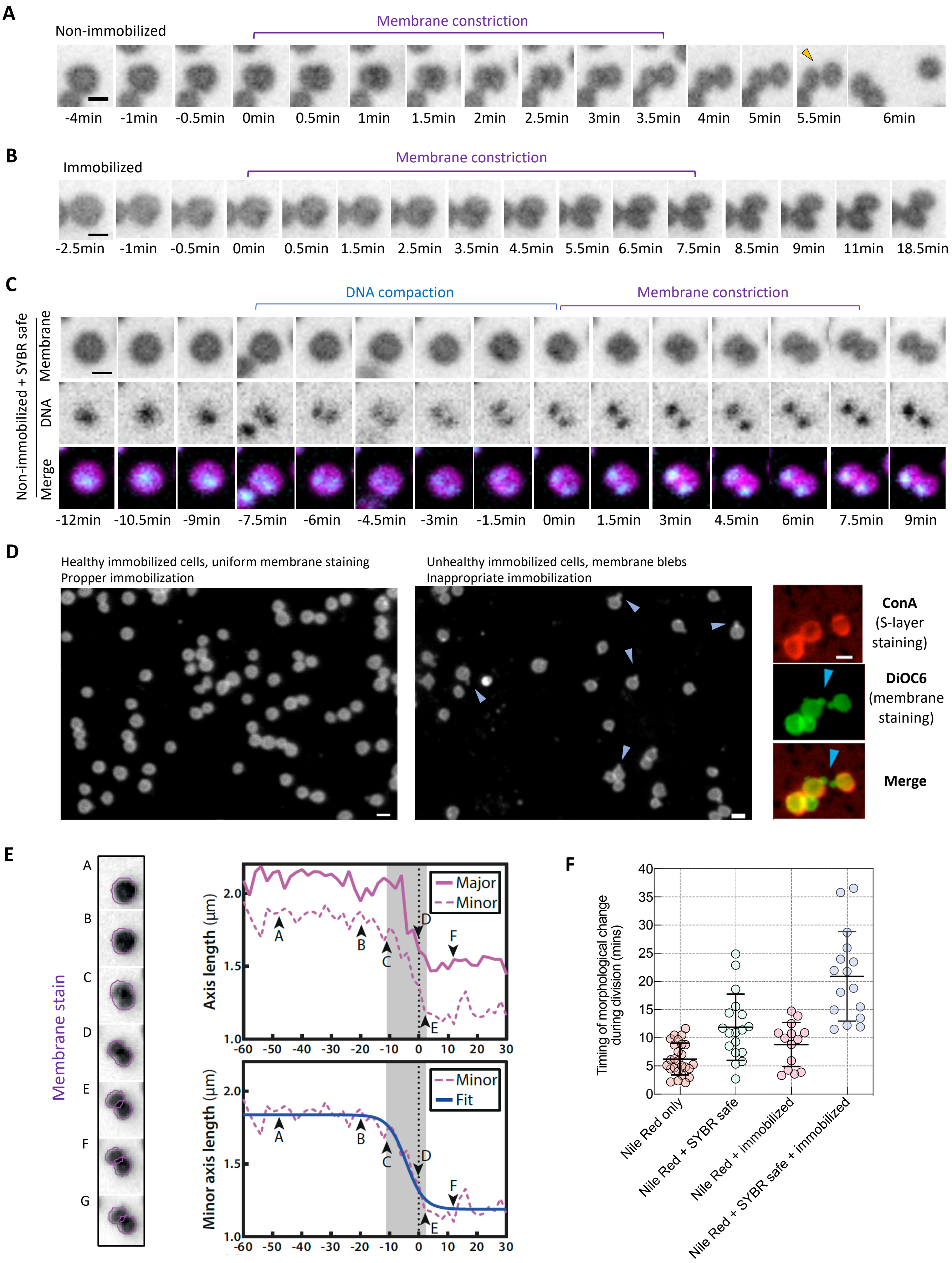

Figure S1. *Sulfolobus acidocaldarius* live-imaging in different conditions. Related to Figure 2. (A) Time-lapse imaging of a non-immobilized *Sulfolobus* cell stained with Nile-red. Orange arrowhead = separation of cells. (B) Time-lapse imaging of an immobilized *Sulfolobus* cell stained with Nile-red. (C) Time-lapse imaging of a non-immobilized *Sulfolobus* cell stained with Nile red and SYBR safe (D) Images show *S. acidocaldarius* cells at 75°C to illustrate the

differences between healthy cells and cells stressed by severe immobilization under a pad. Stressed cells typically exhibit blebs (lacking an S-layer) and membrane patches. Stress can be caused by a failure to clean coverslips and/or by unchecked compression. (E) Analysis of cell shape transitions during cell division as measured by changes in the major and minor axis. (F) The impact of different conditions on the timing of cell shape transitions during division. Scale bars: 1µm. Error bars show mean and SD.

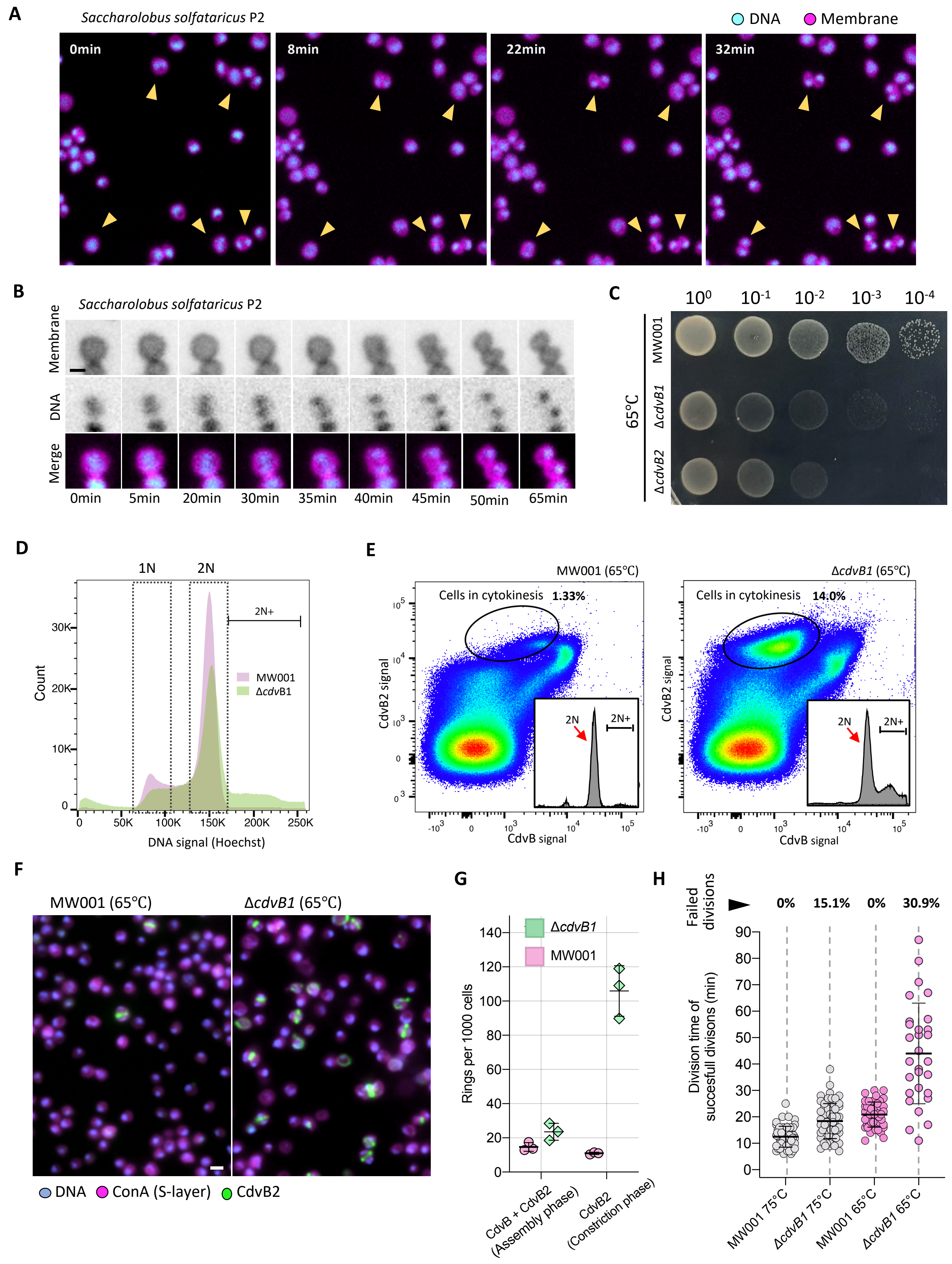

Figure S2. Live imaging of *Saccharolobus solfataricus* and comparative growth of *S. acidocaldarius* MW001 and Δ*cdvB1* at 65°C. Related to Figure 2 and Figure 3. (A) Time-lapse imaging of dividing, immobilized *Saccharolobus solfataricus* P2 cells. (B) Time-lapse imaging of a cell to highlight changes in DNA organization and cell division. (C) Growth of MW001 (background strain), Δ*cdvB1* and Δ*cdvB2* on BNS plates at 65°C, showing that Δ*cdvB1*growth is significantly compromised at low temperatures relative to MW001. (D) Flow cytometry analysis of MW001 and Δ*cdvB1* growth at 65°C, showing an accumulation of >2N cells (DNA content). (E) Flow cytometry analysis showing that

Δ*cdvB1* cells accumulate midway through division (as estimated by using antibodies against CdvB and CdvB2). Moreover, some of these cells have a DNA content of >2N. (F) Immunostaining of MW001 and Δ*cdvB1* cells showing the accumulation of CdvB2 rings in the Δ*cdvB1* strain. (G) Quantification of division rings in MW001 and Δ*cdvB1* at 65°C, showing that cells remain stuck midway through division. (H) Comparison of cell division speeds and failure rates for MW001 and Δ*cdvB1* at both 75°C and 65°C (immobilized cells). Cells from two independent movies were used. Division time was estimated as the duration from the start of constriction until the two cells were physically separated. The total number of cells evaluated were: MW001 75°C (n=60), Δ*cdvB1* 75°C (n=66), MW001 65°C (n=48), Δ*cdvB1* 65°C (n=43). Scale bars: 1μm. Error bars show mean and SD.

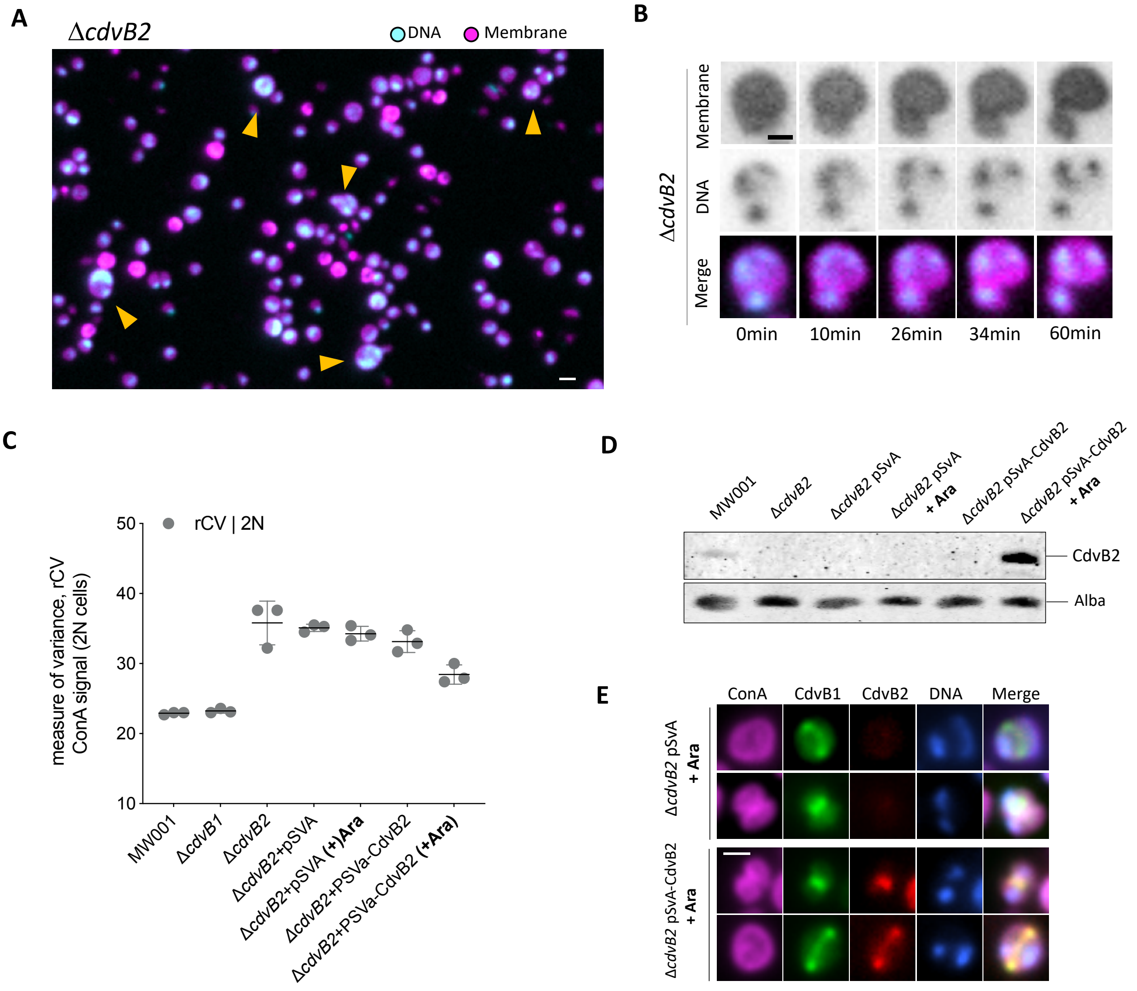

Figure S3. Accumulation of 2N+ cells in Δ*cdvB*2 cells and controls for the Δ*cdvB*2 rescue experiments. Related to Figure 4. (A) Image of Δ*cdvB2* cells at 75°C showing large >2N cells. (B) Time-lapse of an immobilized cell dividing with multiple copies of the genome. Divisions of such large cells are rarely observed, suggesting that they are unlikely to survive for long. (C) 2N cell size variation, estimated by flow cytometry in fixed cells. (D) Western blot showing the expression of CdvB2 from a plasmid after the addition of the inducer L-arabinose. (E) Immunostaining of the Δ*cdvB2*+pSVA-CdvB2 and Δ*cdvB2*+pSVA (empty plasmid) showing that after the induction CdvB2, the formation of CdvB2 rings is restored. (Scale bars: 1μm. rCV = Robust coefficient of variance. Error bars show mean and SD.
